# Pharmacological Modulation of the Kynurenine Pathway Using PF-04859989 Modulates PACAP Signaling in Migraine-Relevant Trigeminal Sensitization

**DOI:** 10.3390/biom16081128

**Published:** 2026-08-03

**Authors:** Evelin Vágvölgyi-Sümegi, Gábor Nagy-Grócz, Zsolt Galla, Edina Katalin Cseh, Zoltán István Tapody, Péter Monostori, János Tajti, Péter Klivényi, László Vécsei, Tamás Körtési

**Affiliations:** 1 Department of Theoretical Health Sciences and Health Management, Faculty of Health Sciences and Social Studies, University of Szeged, Temesvári krt. 31, H-6726 Szeged, Hungary; vagvolgyi-sumegi.evelin@szte.hu (E.V.-S.); nagy-grocz.gabor@szte.hu (G.N.-G.); h162152@gmail.com (Z.I.T.); 2 HUN-REN-SZTE Neuroscience Research Group, Hungarian Research Network, Danube Neuroscience Research Laboratory, University of Szeged, Tisza Lajos krt. 113, H-6725 Szeged, Hungary; klivenyi.peter@med.u-szeged.hu (P.K.); vecsei.laszlo@med.u-szeged.hu (L.V.); 3 Preventive Health Sciences Research Group, Incubation Competence Centre of the Centre of Excellence for Interdisciplinary Research, Development and Innovation, University of Szeged, H-6720 Szeged, Hungary; 4 Metabolic and Newborn Screening Laboratory, Department of Pediatrics, University of Szeged, Korányi Fasor 14-15, H-6720 Szeged, Hungary; gallazs87@gmail.com (Z.G.); monostoripeter@gmail.com (P.M.); 5 Department of Neurology, Albert Szent-Györgyi Faculty of Medicine and Clinical Center, University of Szeged, H-6725 Szeged, Hungary; csehedina.k@gmail.com (E.K.C.); tajti.janos@med.u-szeged.hu (J.T.)

**Keywords:** migraine, neuropeptides, PACAP, kynurenine pathway, kynurenic acid, kynurenine aminotransferase II

## Abstract

Background: Migraine is a disabling neurological disorder in which neuropeptides, particularly pituitary adenylate cyclase-activating polypeptide (PACAP), play pathogenic roles. The kynurenine pathway (KP) is increasingly implicated in migraine-related glutamatergic and neuroinflammatory mechanisms. Previously, we showed that the neuroprotective metabolite kynurenic acid attenuates PACAP overexpression following trigeminovascular activation. This study investigated the effects of kynurenine aminotransferase II (KAT-II) inhibition on PACAP expression and KP metabolites during trigeminal sensitization. Methods: Rats received Complete Freund’s Adjuvant (CFA) or saline injection into the right whisker pad. KAT-II inhibitor PF-04859989 (16 and 32 mg/kg) or saline was administered intraperitoneally 72 h later. Blood samples and nucleus trigeminus caudalis (TNC) were collected for PACAP and KP quantification. Results: CFA treatment induced significant PACAP overexpression in the TNC and altered peripheral KP metabolite levels. PF-04859989 further increased PACAP expression, reaching significance at the 16 mg/kg dose, whereas no significant additional effect was observed at 32 mg/kg. In parallel, treatment with PF-04859989 altered peripheral KP metabolite concentrations in the peripheral inflammatory model, predominantly at the lower dose. Conclusions: This study demonstrates that KAT-II inhibition by PF-04859989 modulates PACAP signaling and KP metabolism under trigeminal inflammatory conditions. These findings support KP–PACAP interactions and may identify novel migraine-related therapeutic targets.

## 1. Introduction

Migraine is a leading contributor to headache disorders, which collectively represent the second leading cause of years lived with disability (YLD) worldwide and impose a substantial individual and socioeconomic burden [1]. Despite extensive research, the pathophysiology of migraine remains incompletely understood, with current evidence supporting a multifactorial process involving genetic susceptibility, neurovascular dysfunction, altered neurotransmission, and inflammatory mechanisms [2,3].

Neurogenic inflammation and trigeminal sensitization are well-established core mechanisms in migraine pathophysiology, characterized by sustained activation of trigeminovascular pathways, peripheral and central sensitization processes, and the release of pro-inflammatory and pro-nociceptive mediators within both peripheral and central nervous system structures. These processes are mediated by multiple neurochemical signaling systems that contribute to the initiation and maintenance of migraine attacks [4,5]. Among the factors contributing to migraine, neuropeptides are key players in the mechanisms that initiate and maintain attacks. In particular, calcitonin gene-related peptide (CGRP) and pituitary adenylate cyclase-activating polypeptide (PACAP) have been highlighted for their potent vasodilatory, pro-nociceptive, and pro-inflammatory effects in migraine-relevant pathways [6,7,8]. CGRP-targeted therapies, including monoclonal antibodies and receptor antagonists, have proven effective in migraine, underscoring the therapeutic relevance of neuropeptides [9]. Although PACAP is strongly implicated in migraine, its therapeutic potential is still under investigation. Clinical trials of PACAP-targeting antibodies have shown promising reductions in monthly migraine days, but larger confirmatory studies are still ongoing [10,11].

PACAP is widely expressed throughout the central and peripheral nervous systems, including key components of the trigeminovascular pathway [12,13]. In our previous study, we demonstrated that trigeminal activation induced by Complete Freund’s Adjuvant (CFA) results in a simultaneous upregulation of CGRP and PACAP expression in the brainstem, and represents a well-established model of trigeminal sensitization relevant to migraine research [14]. Moreover, the observed correlations between increased neuropeptide expression and decreased mechanical pain thresholds provided direct evidence for the contribution of neuropeptides to migraine-associated hyperalgesia. These experimental findings are consistent with observations that PACAP administration triggers migraine-like responses in various experimental models, and that PACAP expression is increased in different migraine-like animal models [15,16]. Clinically, intravenous administration of PACAP-38 induces delayed migraine-like attacks in patients with migraine, but not in healthy individuals, suggesting a disease-specific hypersensitivity to PACAP signaling [17]. In migraineurs, the expression of PACAP in the peripheral blood is increased during a migraine attack compared with the interictal period [18]. These findings underscore the central role of PACAP in migraine and highlight its potential as a therapeutic target.

Beyond neuropeptide studies, growing attention in headache research has focused on mechanisms that modulate excitatory neurotransmission and neuroinflammation. The kynurenine pathway (KP), the principal mechanism for tryptophan (TRP) catabolism, produces several biologically active metabolites capable of influencing neural activity and function [19]. A schematic overview of the major tryptophan metabolic pathways, including the KP, the principal metabolites, and the enzymes involved, is provided in Supplementary Figure S1. Among the KP metabolites, kynurenic acid (KYNA) and quinolinic acid (QUIN) are major neuroactive metabolites with opposing effects on glutamatergic neurotransmission. While KYNA acts as an endogenous N-methyl-D-aspartate (NMDA) receptor antagonist, QUIN functions as an endogenous NMDA receptor agonist [20,21]. KYNA is synthesized from L-kynurenine (L-KYN) by kynurenine aminotransferases (KAT), with KAT-II being the predominant isoform in the central nervous system, whereas peripheral KYNA synthesis involves the coordinated activity of multiple KAT isoforms [22]. Dysregulation of glutamatergic signaling is a well-established feature of migraine pathophysiology. Cortical spreading depression (CSD), which forms the electrophysiological basis of migraine aura, is crucially dependent on excessive glutamate release and NMDA receptor activation [23,24]. Consequently, mechanisms regulating kynurenine metabolism may have profound effects on migraine susceptibility through the modulation of excitatory neurotransmission. In experimental migraine-like conditions, elevated levels of glutamate, L-KYN, and KYNA were observed in central pain pathways, indicating early activation of excitatory and compensatory anti-excitatory mechanisms [25]. Similarly, clinical data have demonstrated a marked shift in peripheral kynurenine metabolism in migraine patients, indicating an extensive imbalance in TRP catabolism [26].

In our previous study, we demonstrated that KYNA and its synthetic analogue are able to attenuate PACAP overexpression under migraine-like conditions [27]. This observation provided the first indication of a functional interaction between the KP and PACAP signaling in migraine, although the precise mechanisms underlying this interaction remain to be clarified.

Considering the crucial roles of PACAP and the KP in migraine, together with their enigmatic interaction, pharmacological inhibition of KAT-II was used as an experimental approach to investigate the contribution of endogenous KYNA synthesis to PACAP regulation. Therefore, PF-04859989 was selected as a well-characterized, brain-penetrant, highly selective irreversible KAT-II inhibitor that has been extensively characterized as a pharmacological tool for reducing endogenous brain KYNA levels in vivo [28,29]. The present study aimed to investigate the effects of pharmacological KAT-II inhibition using PF-04859989 on PACAP expression and KP metabolism in an experimental model of trigeminal sensitization.

## 2. Materials and Methods

### 2.1. Animals

Thirty young adult (10–12 weeks old) male Sprague–Dawley rats were used in this study. The animals were bred and maintained under standard laboratory conditions, with a 12-h light/dark cycle at 24 ± 1 °C and approximately 50% relative humidity in the Laboratory Animal House of the Department of Neurology. Rats had free access to standard chow and water.

### 2.2. Ethics

All experimental procedures were conducted in full compliance with Act 1998/XXVIII of the Hungarian Parliament on Animal Experiments (243/1988) and with the recommendations of the International Association for the Study of Pain and the European Communities Council (86/609/EEC). The study adhered to the Ethical Codex of Animal Experiments and was approved by the Animal Welfare Committee of the University of Szeged (Approval No. XI./1235/2025). The experimental design complied with the principles of the 3Rs (replacement, reduction, and refinement), and sample size estimation was performed a priori based on one-way analysis of variance (ANOVA), ensuring the use of the minimum number of animals required for statistically valid analysis.

### 2.3. Drugs

Complete Freund’s Adjuvant (CFA) killed mycobacteria suspended in paraffin oil, 1 mg/mL was obtained from Sigma-Aldrich (St. Louis, MO, USA) and 50 μL was administered per animal. The KAT-II inhibitor PF-04859989 was also acquired from Sigma-Aldrich and administered intraperitoneally at doses of 16 mg/kg and 32 mg/kg.

### 2.4. Experimental Protocol

**Figure 1:**
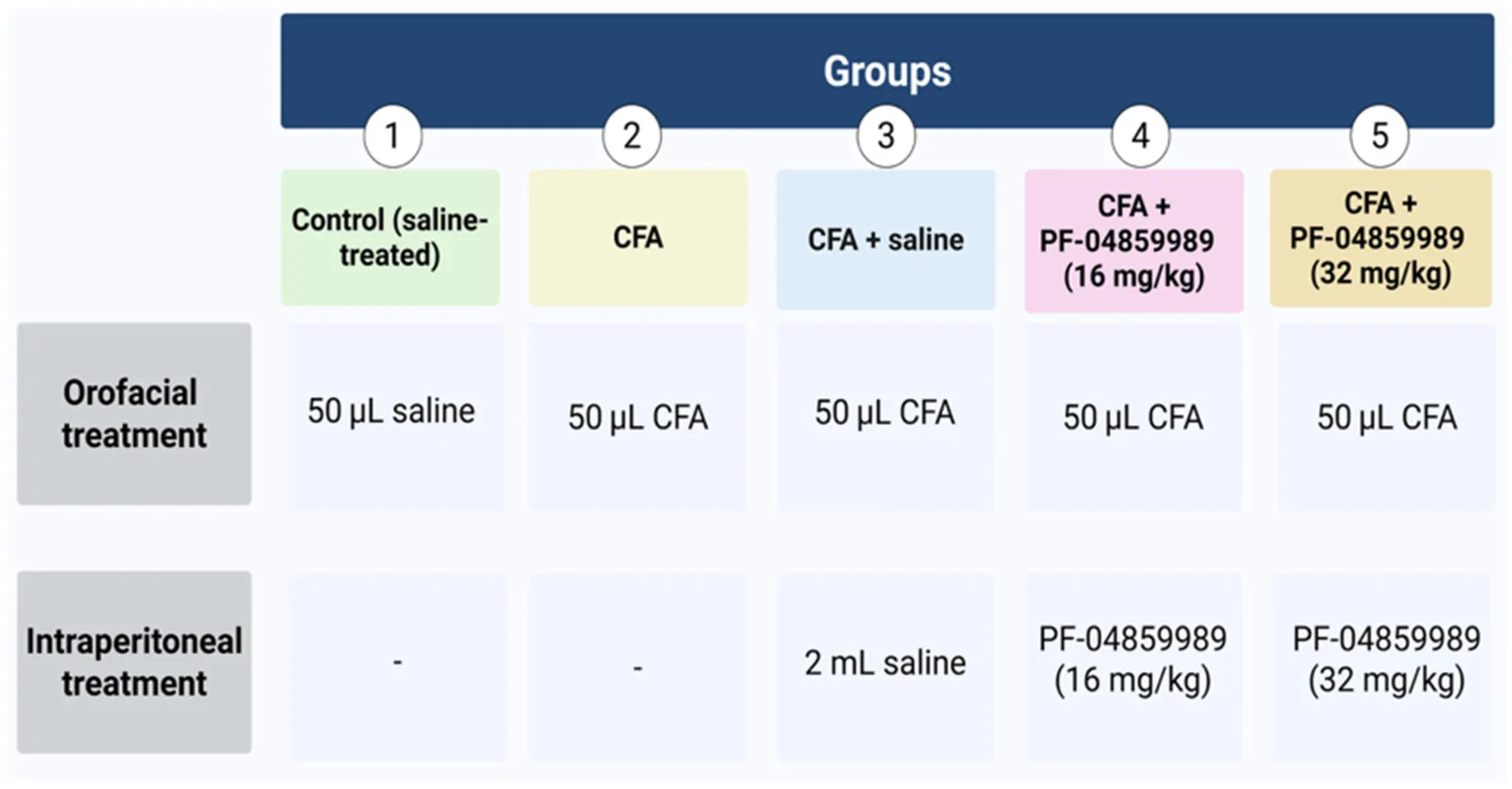
Overview of experimental groups and treatments. The schematic summarizes the orofacial and i.p. treatments applied in each experimental group. Animals were assigned to five experimental groups: saline-treated control, orofacial CFA-treated, orofacial CFA-treated receiving i.p. saline, and orofacial CFA-treated animals receiving the KAT-II inhibitor PF-04859989 at two different doses (16 mg/kg or 32 mg/kg). Orofacial treatments consisted of 50 μL saline or CFA, as appropriate. The i.p. treatments included saline (2 mL) or PF-04859989 administered at the indicated doses. CFA: Complete Freund’s Adjuvant; i.p.: intraperitoneal; KAT-II inhibitor: kynurenine aminotransferase II inhibitor.

Five experimental groups were established, including an orofacial saline-treated control group, an orofacial CFA-treated group, an orofacial CFA-treated group receiving intraperitoneal (i.p.) saline, and two orofacial CFA-treated groups receiving intraperitoneally administered PF-04859989 at doses of 16 or 32 mg/kg. Experimental groups and treatments are presented in Figure 1.

**Figure 2:**
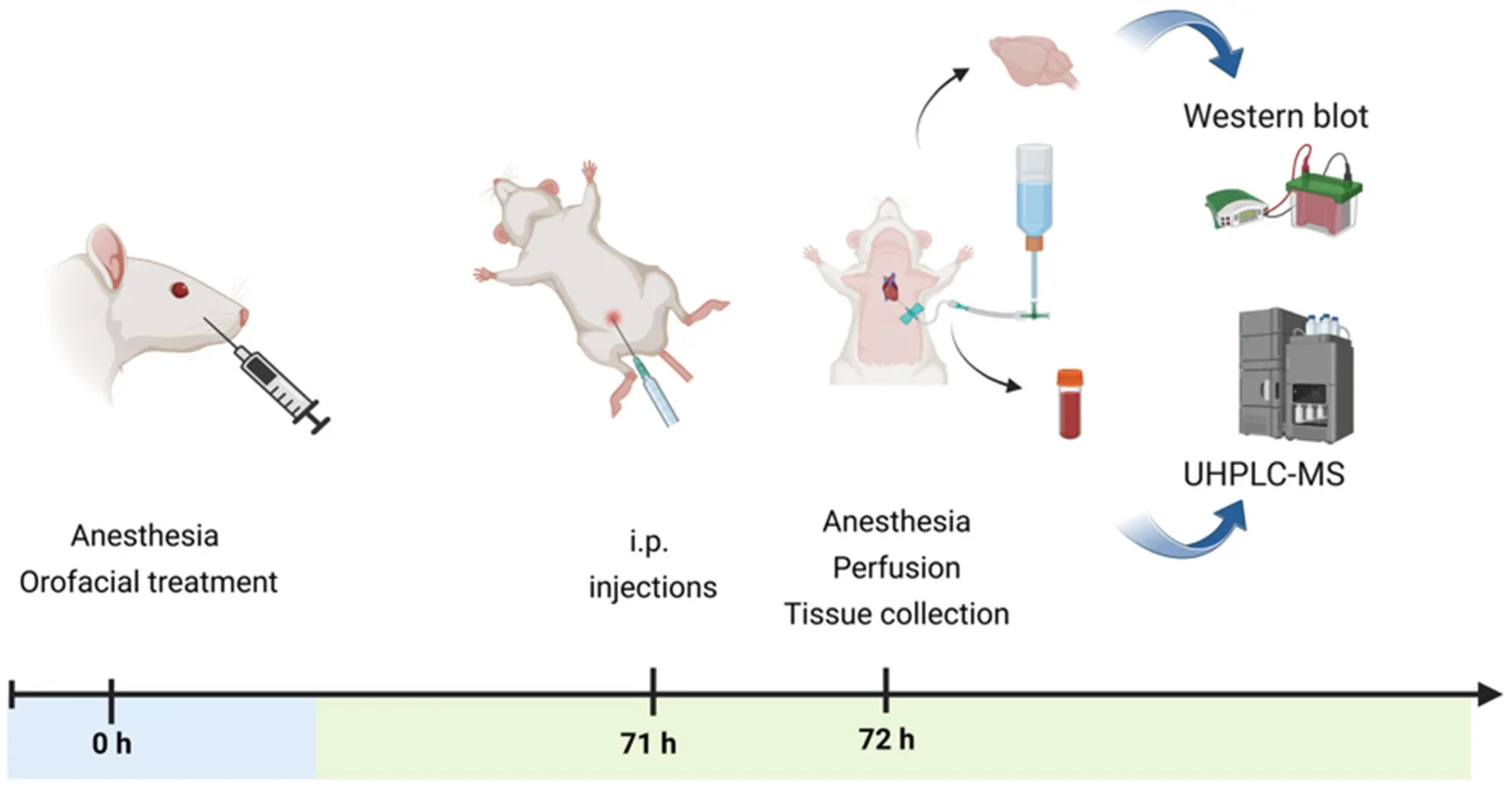
Experimental design of the study. Orofacial CFA administration was performed on Day 0 under anesthesia. The i.p. injections were administered on Day 3. At 71–72 h, animals were anesthetized, followed by transcardial perfusion and tissue and plasma collection. Collected samples were subsequently processed for Western blot and UHPLC-MS analyses. The schematic illustrates the sequence and timing of experimental procedures. UHPLC-MS: ultra-high-performance liquid chromatography-mass spectrometry; CFA: Complete Freund’s Adjuvant; i.p.: intraperitoneal.

Rats were anesthetized with i.p. 4% chloral hydrate solution (10 mL/kg body weight) prior to CFA administration and again before terminal perfusion and tissue collection. The depth of anesthesia was continuously monitored throughout the procedures by repeated assessment of pedal withdrawal and corneal reflexes. Subsequently, 50 μL of CFA was injected into the right whisker pad; control animals received an equal volume of saline. Transcardial perfusion with 200 mL of phosphate-buffered saline (PBS) was performed 72 h after injection, under deep anesthesia induced by i.p. administration of 4% chloral hydrate solution (10 mL/kg body weight)*,* based on previous data showing that the expression of migraine-related neuropeptides peaks at this time point [14]. Saline or the KAT-II inhibitor PF-04859989 dissolved in saline was administered i.p. 1 h before perfusion at doses of 16 or 32 mg/kg, respectively, with a final injection volume of 2 mL. Blood was collected from the left ventricle into ethylenediaminetetraacetic acid (EDTA)-containing tubes, and plasma was obtained by centrifugation. The trigeminal nucleus caudalis (TNC; 1 mm rostral to 5 mm caudal from the obex) was dissected from a bilaterally pooled medullary segment. Tissue samples were stored at −80 °C until further analysis. Expression of preproPACAP, the precursor of PACAP-38, in brain tissues was determined by Western blot, while blood samples were analyzed for KP metabolites using ultra-high-performance liquid chromatography-mass spectrometry (UHPLC-MS). Experimental conditions of the study are presented in Figure 2.

### 2.5. Randomization and Blinding

Animals were randomly allocated to experimental groups using a randomization number generator to minimize potential bias. Group allocation was performed prior to orofacial treatment. All experimental treatments were therefore assigned in advance. Investigators responsible for UHPLC-MS and Western blot analysis and quantification were blinded to group allocation during sample processing, data acquisition, and data analysis. Group identities were only revealed after the completion of all analyses.

### 2.6. Quantitative Determination of preproPACAP Protein Expression by Western Blot Analysis

Tissue samples were sonicated in ice-cold buffer containing 50 mM Tris-HCl, 150 mM NaCl, 0.1% Igepal, 0.1% cholic acid, 2 mg/mL leupeptin, 2 mM phenylmethylsulfonyl fluoride, 1 mg/mL pepstatin, 2 mM EDTA, and 0.1% sodium dodecyl sulfate (SDS). Homogenates were centrifuged at 12,000 rpm for 10 min at 4 °C, and supernatants were aliquoted and stored at −20 °C. Protein concentrations were determined using the bicinchoninic acid (BCA) Protein Assay Kit with bovine serum albumin (BSA) as a standard, and equal amounts of total protein (20 μg per lane) were loaded for sodium dodecyl sulfate-polyacrylamide gel electrophoresis (SDS-PAGE) on 15% Tris-Glycine gels and transferred onto Amersham Protran Premium nitrocellulose membranes (0.20 μm). PageRuler Prestained Protein Ladder (Thermo Fisher Scientific, Waltham, MA, USA; Product No. 26616, 10–180 kDa) was used to estimate molecular weights. Membranes were blocked for 1 h at room temperature in Tris-buffered saline with Tween 20 (TBST) containing 5% nonfat dry milk and then incubated overnight with primary antibodies: PACAP-27/38 rabbit polyclonal antibody (Bioss Antibodies, Woburn, MA, USA; Cat. No. bs-0190R, 1:1000) and horseradish peroxidase-conjugated glyceraldehyde-3-phosphate dehydrogenase (GAPDH) mouse monoclonal antibody (Cell Signaling Technology, Danvers, MA, USA; Cat. No. 51332S; 1:2000). Membranes were subsequently incubated for 2 h at room temperature with horseradish peroxidase-conjugated goat anti-rabbit secondary antibody (Invitrogen, Carlsbad, CA, USA; Cat. No. 31460; 1:5000) in TBST containing 1% nonfat dry milk. Samples were randomly assigned to gels and lanes and processed under blinded conditions to reduce positional effects during electrophoresis. Protein bands were visualized using the Syngene PXi 6 Access Touch Gel Documentation System (Syngene, Cambridge, UK).

### 2.7. Quantitative Determination of TRP Metabolites by UHPLC-MS

All reagents and chemicals were of analytical or liquid chromatography-mass spectrometry grade. TRP and its metabolites and their deuterated forms: d5-tryptophan, d4-kynurenine, d5-kynurenic acid, d4-xanthurenic acid, d3-3-hydroxyanthranilic acid, and d3-quinolinic acid were purchased from Toronto Research Chemicals (Toronto, ON, Canada). The d3-3-hydroxykynurenine was obtained from Buchem B. V. (Apeldoorn, The Netherlands). Acetonitrile was provided by Molar Chemicals (Halásztelek, Hungary). Methanol (MeOH) was purchased from LGC Standards (Wesel, Germany). Formic acid (FA) and water were obtained from VWR Chemicals (Monroeville, PA, USA). The UHPLC-MS/MS system consisted of a PerkinElmer Flexar UHPLC system (two FX-10 binary pumps, solvent manager, autosampler and thermostatic oven, all PerkinElmer Inc. (Waltham, MA, USA), coupled to an AB SCIEX QTRAP 5500 MS/MS triple quadrupole mass spectrometer and controlled by Analyst 1.7.1 software (both AB Sciex, Framingham, MA, USA). TRP and its metabolites were measured in plasma according to a previously published protocol by UHPLC-MS [30].

### 2.8. Statistical Analysis

The Shapiro–Wilk test was used to assess data distribution, complemented by visual inspection of histograms and quantile–quantile (Q-Q) plots. All analyses were performed using independent biological replicates. Each experimental group consisted of n = 6 independent biological replicates derived from separate animals. Data of the Western blot analysis followed a normal distribution, so after the one-way ANOVA test, we used the Fisher’s least significant difference (LSD) post hoc test to analyze the results. For UHPLC-MS analysis, data showing a normal distribution were analyzed using one-way ANOVA followed by Dunnett’s post hoc test, whereas data that did not follow a normal distribution were analyzed using the Mann–Whitney test. A probability level of *p* < 0.05 was considered significant. Data are presented as box-and-whisker plots showing median and interquartile range with minimum and maximum values.

## 3. Results

### 3.1. Differences in Plasma Levels of TRP Metabolites Among Experimental Groups

**Figure 3:**
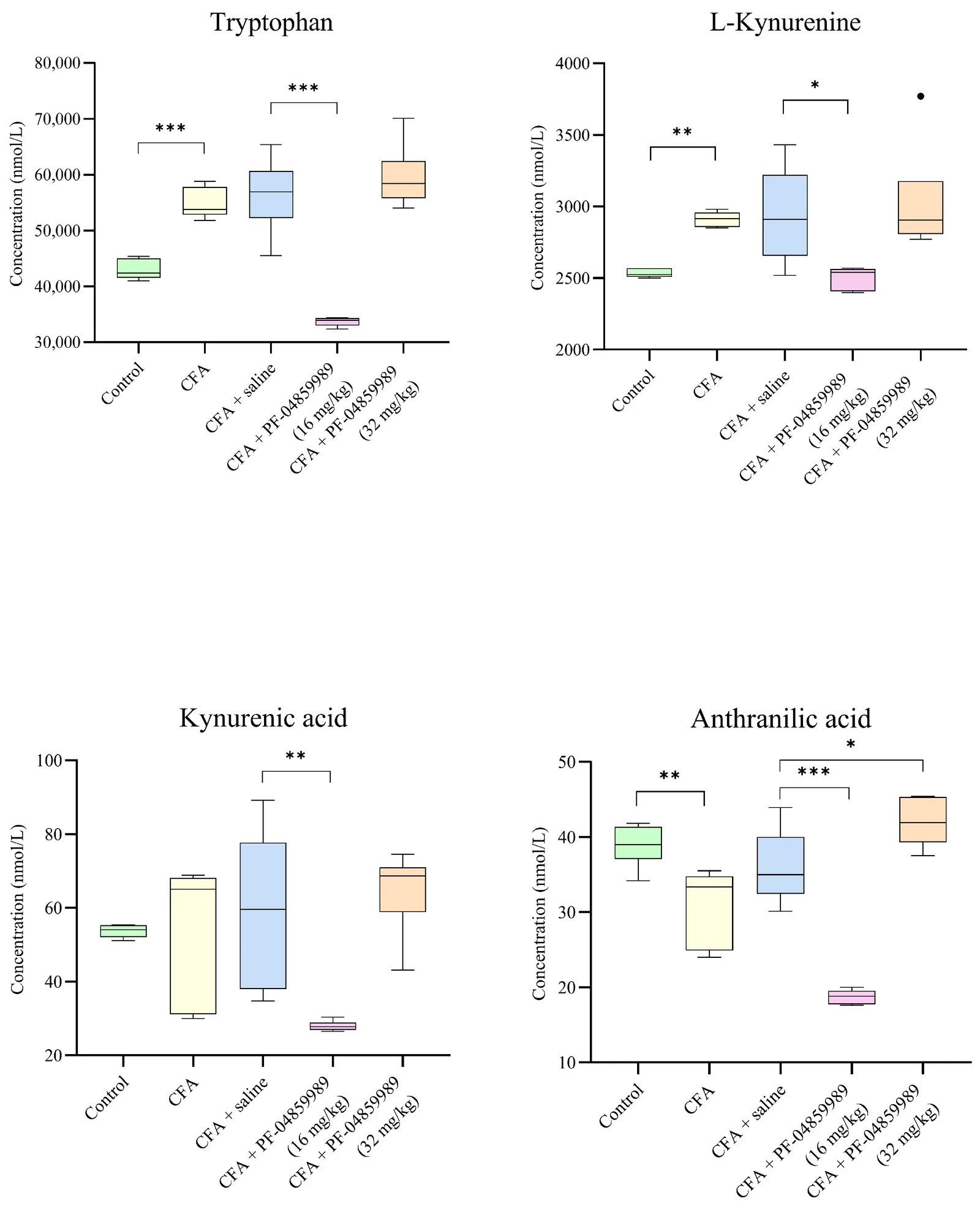
Peripheral plasma concentrations of tryptophan, L-kynurenine, kynurenic acid, and anthranilic acid across experimental groups. Data are presented as boxplots showing the median, interquartile range, and minimum–maximum values, with individual data points overlaid. Black dots represent outliers. Statistical analyses were performed using one-way ANOVA followed by Dunnett’s post hoc test or the Mann–Whitney U test, as appropriate. Compared with the control group, CFA treatment significantly altered the plasma concentrations of TRP (*** *p* < 0.001), L-KYN (** *p* = 0.002), and ANA (** *p* = 0.007). Relative to the CFA + saline group, treatment with PF-04859989 at 16 mg/kg significantly affected the TRP (*** *p* < 0.001), L-KYN (* *p* = 0.022), KYNA (** *p* = 0.002), and ANA (*** *p* < 0.001). In contrast, ANA levels were significantly increased following treatment with PF-04859989 at 32 mg/kg (* *p* = 0.029). CFA: Complete Freund’s Adjuvant; ANOVA: analysis of variance; TRP: tryptophan; L-KYN: L-kynurenine; KYNA: kynurenic acid; ANA: anthranilic acid.

Peripheral plasma concentrations of TRP and its metabolites in the KP were altered in CFA-treated animals compared to controls, with a significant overall group effect observed for TRP levels (partial η^2^ = 0.868). TRP levels were significantly increased following CFA treatment (*p* < 0.001). Administration of PF-04859989 at 16 mg/kg significantly attenuated this CFA-induced increase in TRP levels (*p* < 0.001), whereas no significant differences were observed at the 32 mg/kg dose compared to the CFA + saline group, despite numerically higher TRP levels. Similarly, L-KYN concentrations were significantly increased following CFA treatment (*p* = 0.002). This increase was significantly attenuated by PF-04859989 at 16 mg/kg (*p* = 0.022), whereas no significant changes were detected at the 32 mg/kg dose compared to the CFA + saline group. No significant differences in the KYNA levels were observed between the control and CFA groups. However, treatment with PF-04859989 at 16 mg/kg significantly reduced the KYNA concentrations compared to the CFA + saline group (*p* = 0.002), whereas no significant changes were detected at the 32 mg/kg dose. Anthranilic acid (ANA) levels were significantly reduced in CFA-treated animals receiving PF-04859989 at 16 mg/kg (*p* < 0.001), whereas a significant increase was observed at 32 mg/kg (*p* = 0.029), indicating a non-linear response to the two tested doses. A significant overall group effect was also identified for ANA levels (partial η^2^ = 0.856). Differences in plasma levels of TRP, L-KYN, KYNA, and ANA are presented in Figure 3.

**Figure 4:**
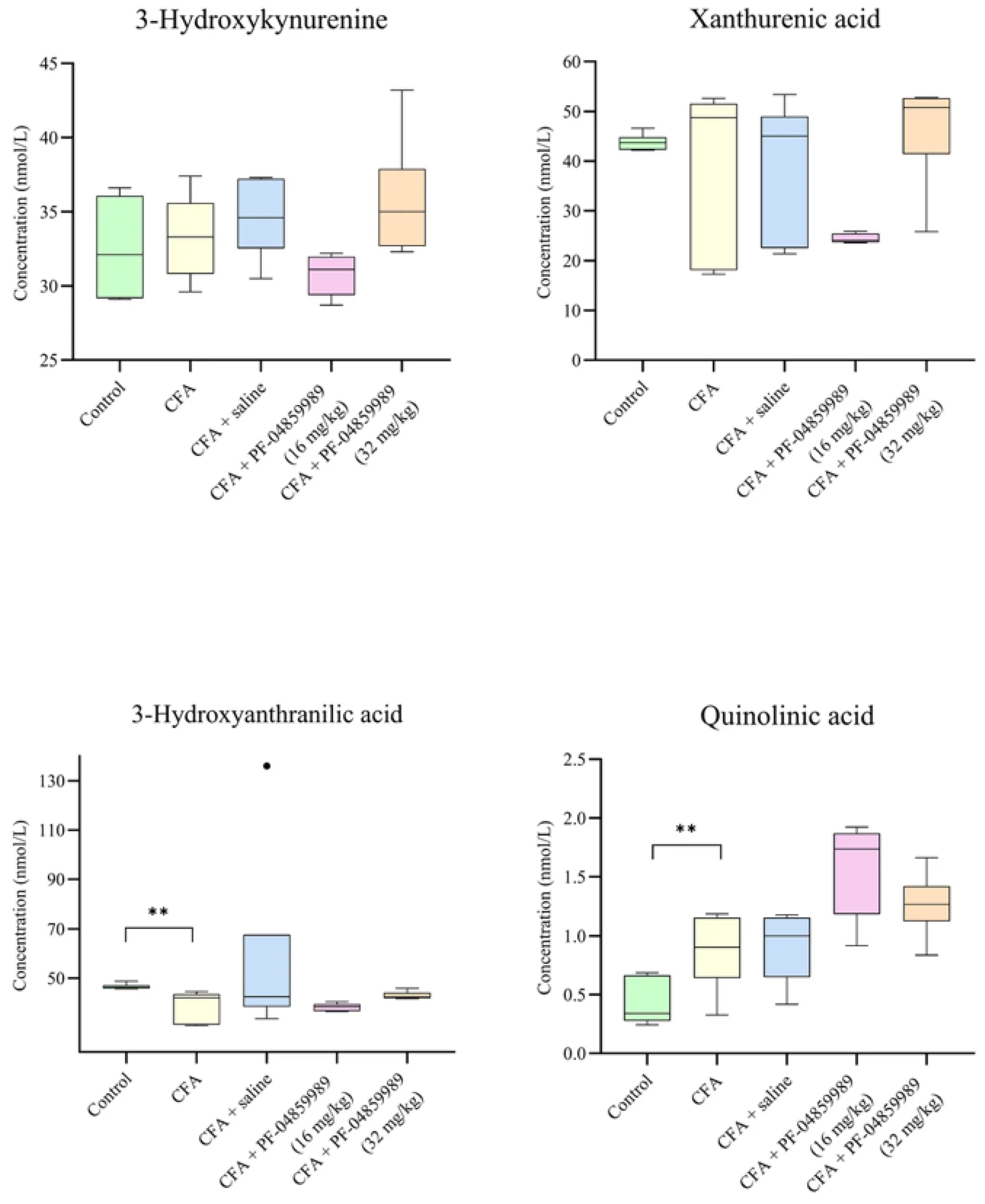
Peripheral plasma concentrations of 3-hydroxykynurenine, xanthurenic acid, 3-hydroxyanthranilic acid, and quinolinic acid across experimental groups. Data are presented as boxplots showing the median, interquartile range, and minimum–maximum values, with individual data points overlaid. Black dots represent outliers. Statistical analyses were performed using one-way ANOVA followed by Dunnett’s post hoc test or the Mann–Whitney U test, as appropriate. Compared with the control group, CFA treatment significantly altered the plasma concentrations of 3-HANA (** *p* = 0.002) and QUIN (** *p* = 0.002). A slight, non-significant decrease in 3-HANA was observed following treatment with PF-04859989 at a dose of 16 mg/kg, with no effect at the higher dose. QUIN showed a non-significant increase after administration of the 16 mg/kg PF-04859989 dose. 3-HK and XA displayed decreasing trends following treatment with PF-04859989 at 16 mg/kg, although these changes did not reach statistical significance. CFA: Complete Freund’s Adjuvant; ANOVA: analysis of variance; 3-HANA: 3-hydroxyanthranilic acid; 3-HK: 3-hydroxykynurenine; XA: xanthurenic acid; QUIN: quinolinic acid.

Peripheral plasma concentrations of 3-hydroxykynurenine (3-HK), xanthurenic acid (XA), 3-hydroxyanthranilic acid (3-HANA), and QUIN showed variable alterations among the experimental groups. A moderate overall group effect was observed for 3-HK levels (partial η^2^ = 0.275). 3-HANA concentrations differed significantly between the control and CFA-treated animals (*p* = 0.002) and a slight, non-significant decrease was observed after the administration of PF-04859989 at 16 mg/kg, with no effect at the higher dose. QUIN levels were also significantly altered between the control and CFA-treated animals (*p* = 0.002), with a non-significant increase following treatment with PF-04859989 at 16 mg/kg. 3-HK and XA exhibited decreasing trends following treatment with PF-04859989 at 16 mg/kg, in line with the overall direction of changes observed in several other metabolites; however, these changes did not reach statistical significance. Differences in plasma levels of 3-HK, XA, 3-HANA, and QUIN are presented in Figure 4. Detailed quantitative data for measured KP metabolites are provided in Supplementary Table S1.

### 3.2. Alterations in the preproPACAP Expression in the TNC

**Figure 5:**
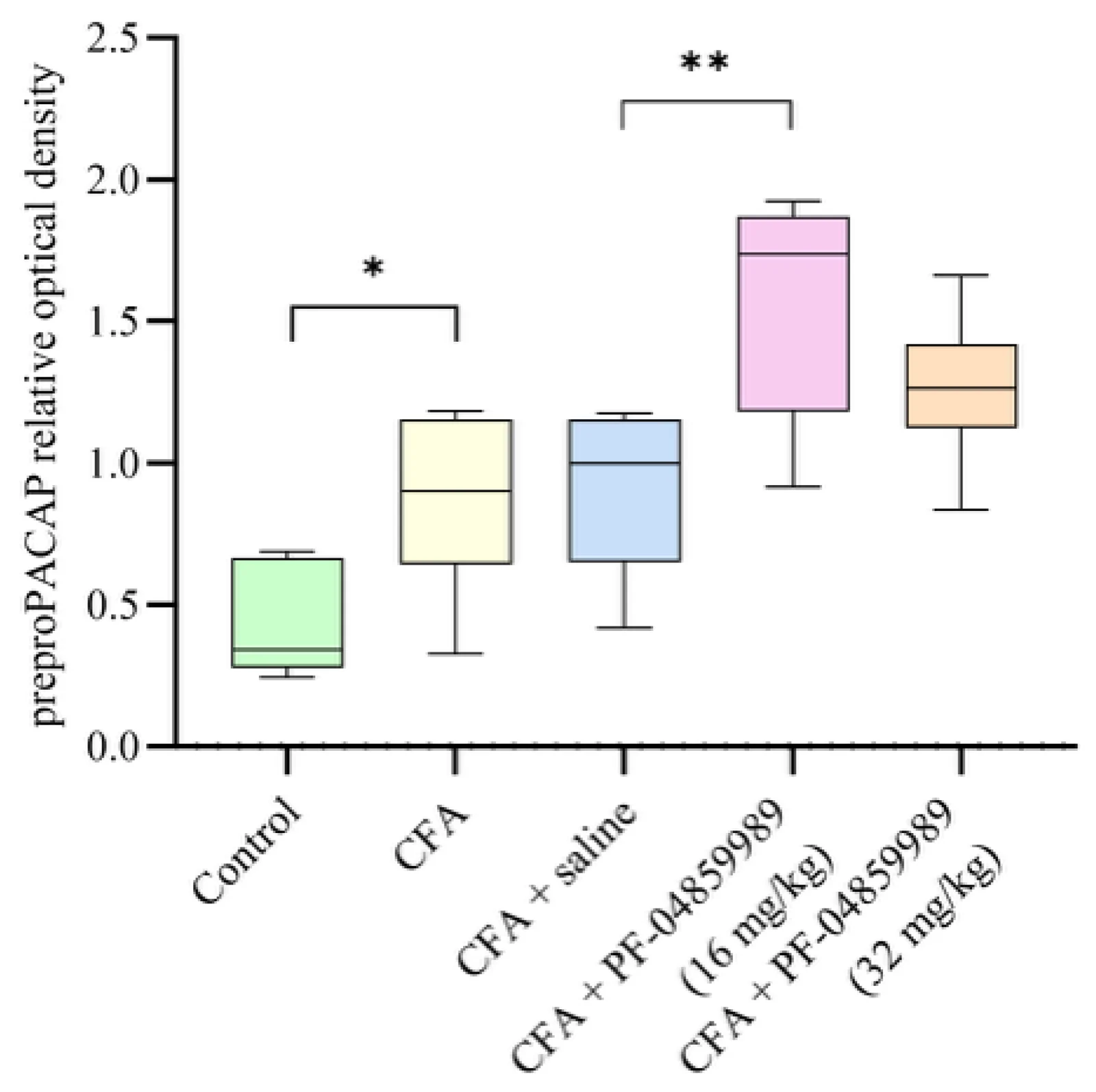
Relative optical density of preproPACAP protein expression across experimental groups, determined by Western blot analysis. Data are presented as boxplots showing the median, interquartile range, and minimum–maximum values, with individual data points overlaid. Black dots represent outliers. Statistical analyses were performed using one-way ANOVA followed by Dunnett’s post hoc test or the Mann–Whitney U test, as appropriate. Compared with the control group, CFA treatment significantly increased preproPACAP expression (* *p* = 0.016). Relative to the CFA + saline group, treatment with PF-04859989 at 16 mg/kg significantly increased preproPACAP expression (** *p* = 0.001). Relative optical density values represent preproPACAP band intensities normalized to GAPDH. CFA: Complete Freund’s Adjuvant; preproPACAP: prepro-pituitary adenylate cyclase-activating polypeptide; GAPDH: glyceraldehyde 3-phosphate dehydrogenase.

**Figure 6:**
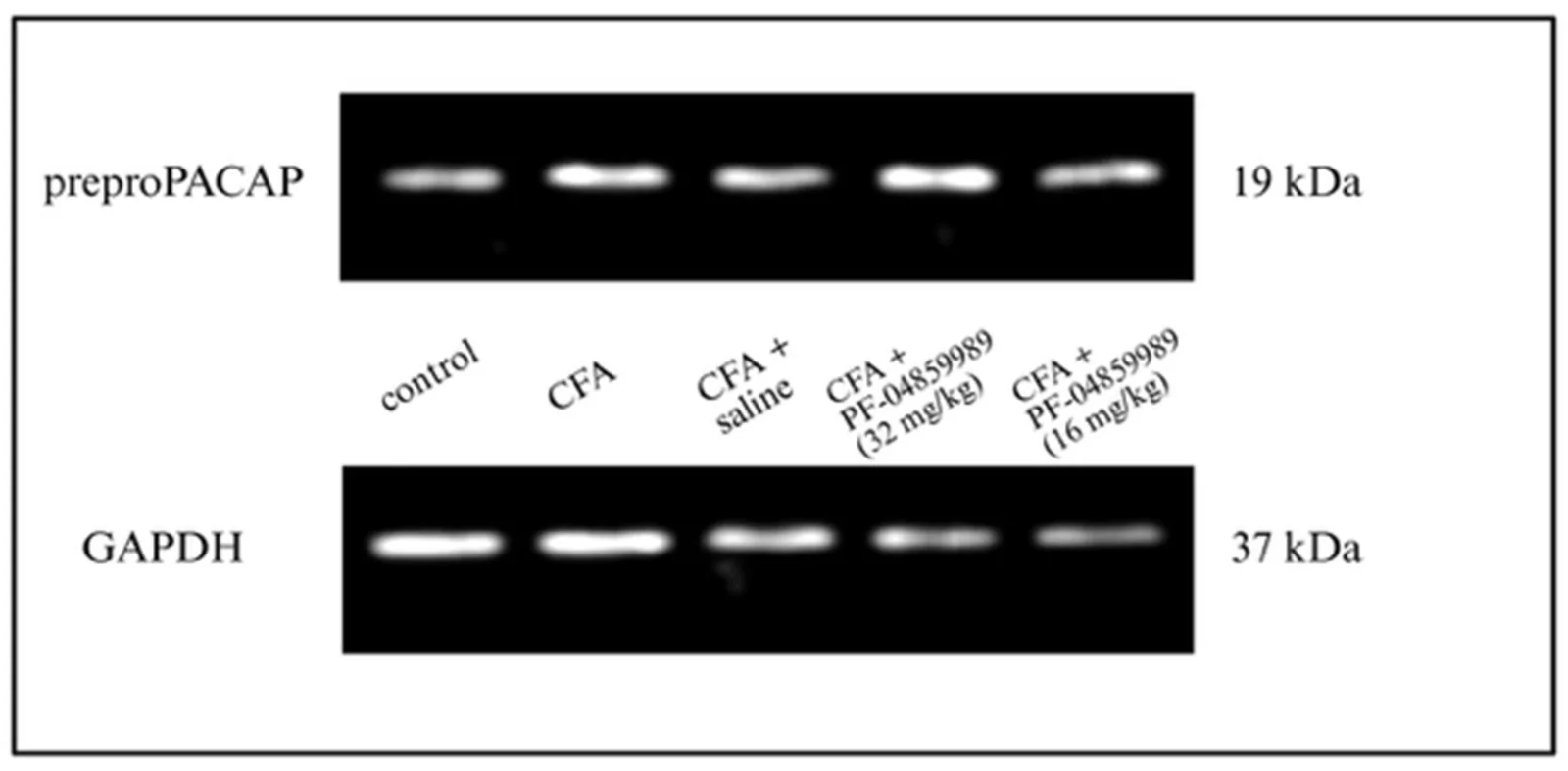
Representative Western blot of preproPACAP with GAPDH loading control in the TNC. CFA: Complete Freund’s Adjuvant; GAPDH: glyceraldehyde 3-phosphate dehydrogenase; preproPACAP: prepro-pituitary adenylate cyclase-activating polypeptide.

A significant overall group effect was observed for preproPACAP expression in the TNC (partial η^2^ = 0.667). The relative optical density of preproPACAP was significantly higher 72 h after CFA treatment in the orofacial CFA-treated group (*p* = 0.017) and in the CFA-treated + i.p. saline group (*p* = 0.01) compared with the control group in the TNC. PreproPACAP expression was significantly elevated in the CFA-treated + PF-04859989 groups at 16 mg/kg (*p* = 0.001) and 32 mg/kg (*p* = 0.031) compared with the CFA-treated group. Compared with the CFA + i.p. saline group, only the 16 mg/kg PF-04859989 treatment induced a significant increase (*p* = 0.001), whereas the 32 mg/kg dose showed no significant difference (*p* = 0.052). Alterations in preproPACAP expression in the TNC are presented in Figure 5, and a representative Western blot image is shown in Figure 6. Detailed quantitative data from the Western blot analyses are provided in Supplementary Table S2.

## 4. Discussion

Migraine research in recent years has increasingly focused on neuropeptides as key pathogenic factors. Among these, PACAP has emerged as a crucial mediator in the pathophysiology of migraine. Beyond this mechanistic role, PACAP has also gained considerable attention as a potential therapeutic target, with ongoing efforts to develop PACAP-targeting therapy [31,32]. In addition to neuropeptides, increasing attention has been directed toward metabolic pathways that regulate excitatory neurotransmission. In this context, the KP plays an important role by modulating glutamatergic signaling, a central mechanism in migraine [33].

The present study was designed to further clarify the relationship between the KP and PACAP signaling under migraine-associated trigeminal sensitization experimental conditions by examining the effects of KAT-II inhibition with PF-04859989. In this study, terminal sampling was performed 3 days after orofacial CFA administration, as our previous experiments indicated that neurochemical alterations and changes in neuropeptide expression are most evident at this time point [14,25]. Our results demonstrated that orofacial CFA administration alters selected KP metabolites and leads to increased PACAP expression. These findings are consistent with selected clinical observations reporting alterations in kynurenine metabolism and neuropeptide expression during migraine attacks [18,26]. A novel aspect of the present study is that administration of PF-04859989 following CFA treatment induced significant changes in KP metabolite levels and a marked increase in PACAP expression.

PF-04859989 was originally characterized as a selective KAT-II inhibitor among the enzymes examined. In the same study, doses between 32 and 100 mg/kg produced a plateau of approximately 80% KAT-II inhibition under the experimental conditions investigated [29]. However, subsequent studies have reported additional molecular interactions, including the inhibition of glutamate oxaloacetate transaminase 1 (GOT1), suggesting that its pharmacological profile may be more complex than originally appreciated [34]. Nevertheless, the functional relevance of these additional interactions in migraine-relevant trigeminal sensitization remains to be established.

Notably, the lower dose of the KAT-II inhibitor induced more pronounced effects on PACAP expression and several KP metabolites than the higher dose. Consistent with this interpretation, opposite effects of the two PF-04859989 doses were observed for ANA, further supporting the complex and non-linear regulation of the KP, although the mechanism underlying this finding remains unclear [35,36]. The present data support a link between these mechanisms and highlight the potential for the indirect modulation of PACAP signaling via KP regulation. In the KP, multiple biologically active metabolites interact within a dynamically regulated metabolic network. Within this system, pharmacological modulation or inhibition may induce adaptive, compensatory responses that partially offset the effects observed at lower doses. In parallel, the functional effects of KP metabolites are strongly context-, and concentration-dependent, which may further contribute to the heterogeneous, dose-dependent responses observed in the present study [19,37].

**Figure 7:**
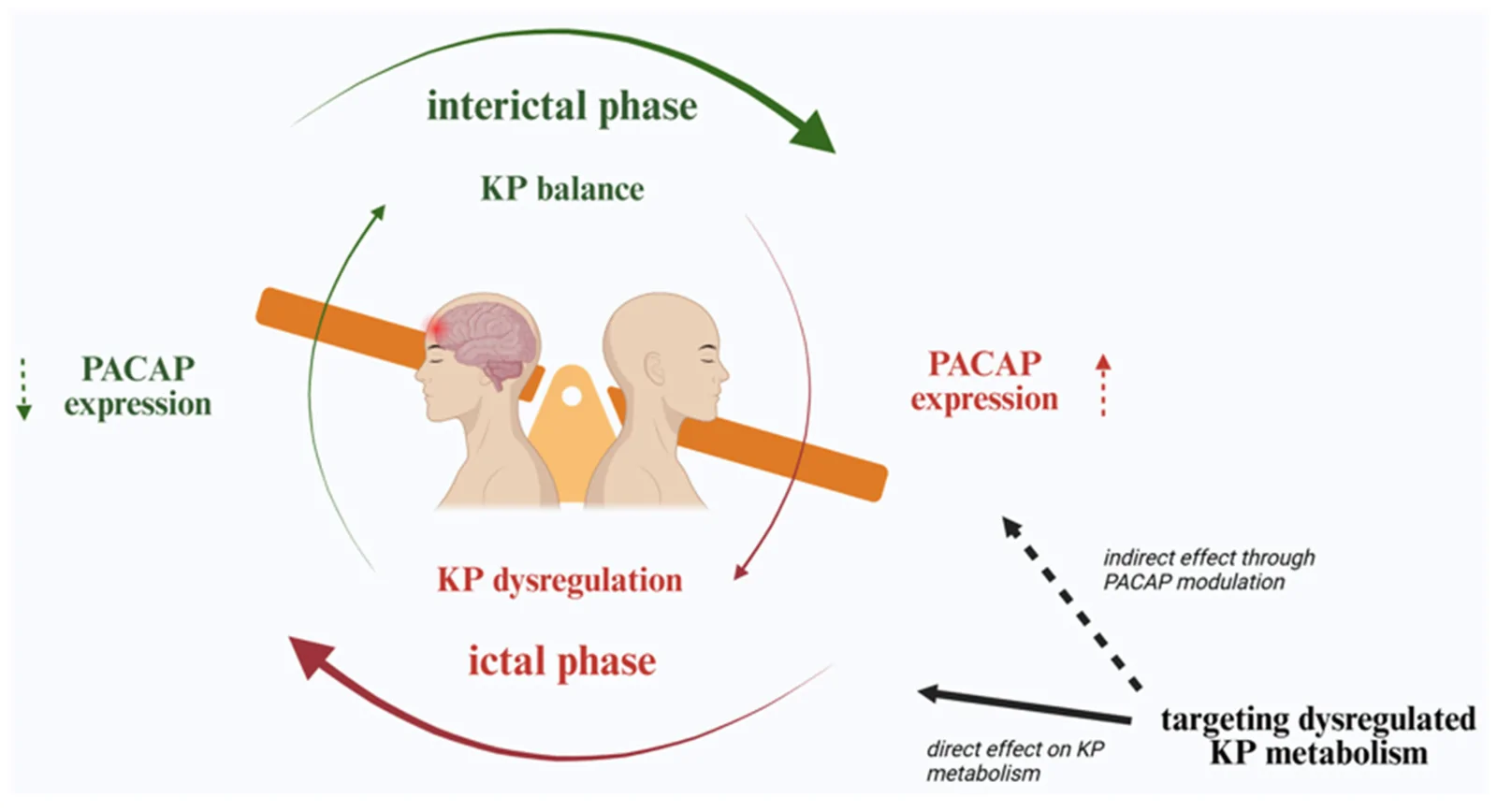
Schematic summary of the proposed relationship between the KP and PACAP in migraine. During the interictal phase of migraine, KP metabolism and PACAP levels are maintained at physiological balance. In the ictal phase, disruption of KP homeostasis is associated with increased PACAP expression and altered neurochemical signaling relevant to migraine mechanisms. Altered KP activity may be linked to changes in PACAP signaling, suggesting a functional interaction between metabolic regulation and neuropeptide-mediated processes. Accordingly, the schematic illustrates the hypothesis that targeting dysregulated KP metabolism may influence migraine-related pathophysiological processes both directly through the modulation of KP metabolism and indirectly through the modulation of PACAP signaling, thereby highlighting its potential therapeutic relevance in migraine. KP: kynurenine pathway; PACAP: pituitary adenylate cyclase-activating polypeptide.

These observations extend the findings of our previous study, in which we demonstrated that exogenous administration of KYNA and its synthetic analogue attenuates PACAP overexpression following trigeminovascular system (TS) activation [27]. The potential link between the KP and PACAP in migraine is illustrated in Figure 7.

Metabolomic measurements of the KP revealed a complex and partially divergent pattern of changes. CFA treatment resulted in increased levels of TRP, L-KYN, and QUIN, accompanied by decreased levels of ANA and 3-HANA, while peripheral KYNA levels remained unchanged. The apparent differences between the present findings and our previous study likely reflect the dynamic temporal regulation of the KP during CFA-induced inflammation, as the two studies examined different time points following CFA administration [25]. However, metabolite alterations were heterogeneous, with several displaying dose-dependent responses and achieving statistical significance primarily at the lower dose of PF-04859989. These findings emphasize the intrinsic complexity of the KP and suggest that its regulation involves coordinated, non-linear metabolic interactions [19,38]. Interestingly, although CFA treatment significantly altered several upstream and branch-point KP metabolites, peripheral KYNA levels were not significantly changed. This observation further supports the concept that inflammatory activation induces a selective rather than uniform modulation of the KP. Given the dynamically regulated nature of the pathway, downstream metabolites such as KYNA may be influenced by compensatory metabolic mechanisms or changes in pathway dynamics under inflammatory conditions [39,40]. In addition, although XA is also generated through KAT-mediated transamination reactions, only a non-significant tendency toward lower levels was observed following PF-04859989 treatment. Therefore, further studies will be required to clarify the potential contribution of xanthurenic acid to the observed biological effects. Given the Janus-faced nature of the KP, this apparent complexity can be interpreted as reflecting the coexistence of metabolites with opposing functional properties. While KYNA is generally considered neuroprotective due to its antagonistic effects on glutamatergic neurotransmission, other metabolites such as QUIN exert neurotoxic and excitatory actions. However, the functional consequences of KP activation are unlikely to be determined solely by the balance between these two metabolites, but rather by the integrated actions of multiple dynamically regulated KP metabolites. Importantly, the effects of individual metabolites may also differ in a dose-, context-, and time dependent manner, further contributing to the complexity of pathway regulation [37]. Consequently, the overall impact of KP modulation is likely determined by the balance among these functionally distinct and dynamically regulated components rather than by the absolute levels of any single metabolite. The heterogeneous changes observed in the present study may therefore reflect a dynamic reorganization of this balance in response to inflammatory and nociceptive challenges.

The investigation of neuropeptides as therapeutic targets has marked a new era in migraine management. Compared to previously applied preventive and acute treatment strategies, the introduction of CGRP-targeting monoclonal antibodies has significantly improved therapeutic efficacy while maintaining a favorable safety profile [41]. Nevertheless, it is important to emphasize that despite the relatively homogeneous clinical presentation of migraine, the underlying molecular mechanisms are highly heterogeneous and complex [42,43,44]. In this context, it should be noted that current therapeutic strategies, including CGRP-targeting treatments, are largely applied without prior assessment of individual CGRP expression levels, even though not all patients exhibit elevated CGRP activity [45,46]. This highlights a key limitation of the current, largely standardized treatment approach and underscores the need for more personalized therapeutic strategies.

Given these limitations, there is a growing need to identify novel therapeutic targets, among which PACAP may represent a particularly promising candidate. Clinical phase trials targeting PACAP are currently ongoing. In this context, the PROCEED study is an adaptive, randomized, double-blind, placebo-controlled phase IIb dose-finding clinical trial evaluating multiple subcutaneous doses of Lu AG09222. Lu AG09222 is a monoclonal antibody that specifically binds to the PACAP ligand, thereby inhibiting its biological activity. The study has also been expanded to include intravenous administration in order to more precisely characterize dose–response relationships. The trial enrolls patients with migraine who have shown inadequate responses to previous preventive therapies. Completion of the study is expected in the first half of 2026, followed by the initiation of phase III trials, which may enable Lu AG09222 to emerge as a novel preventive therapy targeting the PACAP pathway [47]. However, monoclonal antibodies directed against the PAC1 receptor have not demonstrated clinical efficacy, suggesting that targeting the ligand may represent a more effective therapeutic approach. Nevertheless, further studies are required to evaluate long-term safety and clinical applicability [48]. Collectively, PACAP signaling and the KP appear to be functionally related, potentially involving glutamatergic and NMDA receptor-dependent pathways, suggesting a possible interaction between metabolic regulation and neuropeptidergic signaling within the TS [33,49,50]. Considering the involvement of neuropeptides in migraine, modulation of PACAP signaling via the KP may represent a potential direction for further investigation into migraine pathophysiology.

However, several limitations should be acknowledged. The findings are based on an experimental animal model and therefore cannot be directly translated to migraine pathophysiology. Although PF-04859989 treatment modulated both KP metabolites and PACAP expression, the observed relationship remains associative rather than causal. Moreover, PACAP expression was assessed centrally in the TNC, whereas KP metabolites were measured in peripheral plasma, warranting further studies to clarify the mechanistic link between metabolic and neuropeptidergic changes. Finally, the heterogeneous dose-dependent responses observed following PF-04859989 treatment highlight the complexity of pathway regulation and require further investigation.

## 5. Conclusions

In summary, this study provides evidence that treatment with PF-04859989, employed as a pharmacological KAT-II inhibitor, is associated with PACAP overexpression in the central nervous system following orofacial inflammatory trigeminal activation. Following CFA treatment, significant alterations in KP metabolism were observed in plasma, characterized by both increases and decreases in individual metabolites. Pharmacological KAT-II inhibition attenuated several of these alterations, whereas the QUIN levels continued to increase. These findings suggest a possible endogenous metabolic influence on PACAP signaling and indicate that the KP may contribute to the regulation of neuropeptide signaling within the trigeminal system, potentially providing new insights into migraine pathomechanisms and the indirect modulation of PACAP-related pathways.

## Data Availability

All data, materials, and protocols supporting the conclusions of this article are available from the corresponding author upon reasonable request.

## Supplementary Materials

The following supporting information can be downloaded at: https://www.mdpi.com/article/10.3390/biom16081128/s1, Figure S1: Schematic overview of tryptophan metabolism; Table S1: Summary of kynurenine pathway metabolites quantified by UHPLC-MS; Table S2: Summary of preproPACAP expression quantified by Western blot analysis. Material S1: Original images of Western blot analysis in Figure 5 and Figure 6.

## Abbreviations

3-HK	3-Hydroxykynurenine
3-HANA	3-Hydroxyanthranilic acid
ANA	Anthranilic acid
ANOVA	Analysis of variance
BCA	Bicinchoninic acid
BSA	Bovine serum albumin
CFA	Complete Freund’s Adjuvant
CGRP	Calcitonin gene-related peptide
CSD	Cortical spreading depression
EDT	Ethylenediaminetetraacetic acid
FA	Formic acid
GAPDH	Glyceraldehyde-3-phosphate dehydrogenase
GOT1	Glutamate oxaloacetate transaminase 1
i.p.	Intraperitoneal
KAT	Kynurenine aminotransferase
KAT-II	Kynurenine aminotransferase II
KP	Kynurenine pathway
KYNA	Kynurenic acid
L-KYN	L-kynurenine
LSD	Least significant difference
MeOH	Methanol
MS/MS	Tandem mass spectrometry
NMDA	N-Methyl-D-aspartate
PAC1 receptor	Pituitary adenylate cyclase-activating polypeptide type 1 receptor
PACAP	Pituitary adenylate cyclase-activating polypeptide
PACAP-27/38	Pituitary adenylate cyclase-activating polypeptide 27/38
PACAP-38	Pituitary adenylate cyclase-activating polypeptide-38
PBS	Phosphate-buffered saline
preproPACAP	Prepro-pituitary adenylate cyclase-activating polypeptide
QUIN	Quinolinic acid
SDS	Sodium dodecyl sulfate
SDS-PAGE	Sodium dodecyl sulfate-polyacrylamide gel electrophoresis
SD	Standard deviation
TBST	Tris-buffered saline with Tween 20
TNC	Trigeminal nucleus caudalis
TRP	Tryptophan
Tris-HCl	Tris hydrochloride
TS	Trigeminovascular system
UHPLC-MS	Ultra-high-performance liquid chromatography-mass spectrometry
XA	Xanthurenic acid
YLD	Years lived with disability
