# Supplementary material for: Pharmacological Modulation of the Kynurenine Pathway Using PF-04859989 Modulates PACAP Signaling in Migraine-Relevant Trigeminal Sensitization
Source: Biomolecules. 2026 Aug 3;16(8):1128. doi: 10.3390/biom16081128 (PMC13511012; doi:10.3390/biom16081128)

Supplementary Figure S1. Schematic overview of tryptophan metabolism

Approximately 90–95% of tryptophan is metabolized through the kynurenine pathway, whereas smaller proportions are utilized for serotonin synthesis, protein synthesis, and microbiota-derived indole production. The figure illustrates the major metabolic pathways of tryptophan, with emphasis on the kynurenine pathway, including the principal metabolites and key enzymes involved. Metabolites quantified in the present study are highlighted in bold.All abbreviations used in the figure are defined.

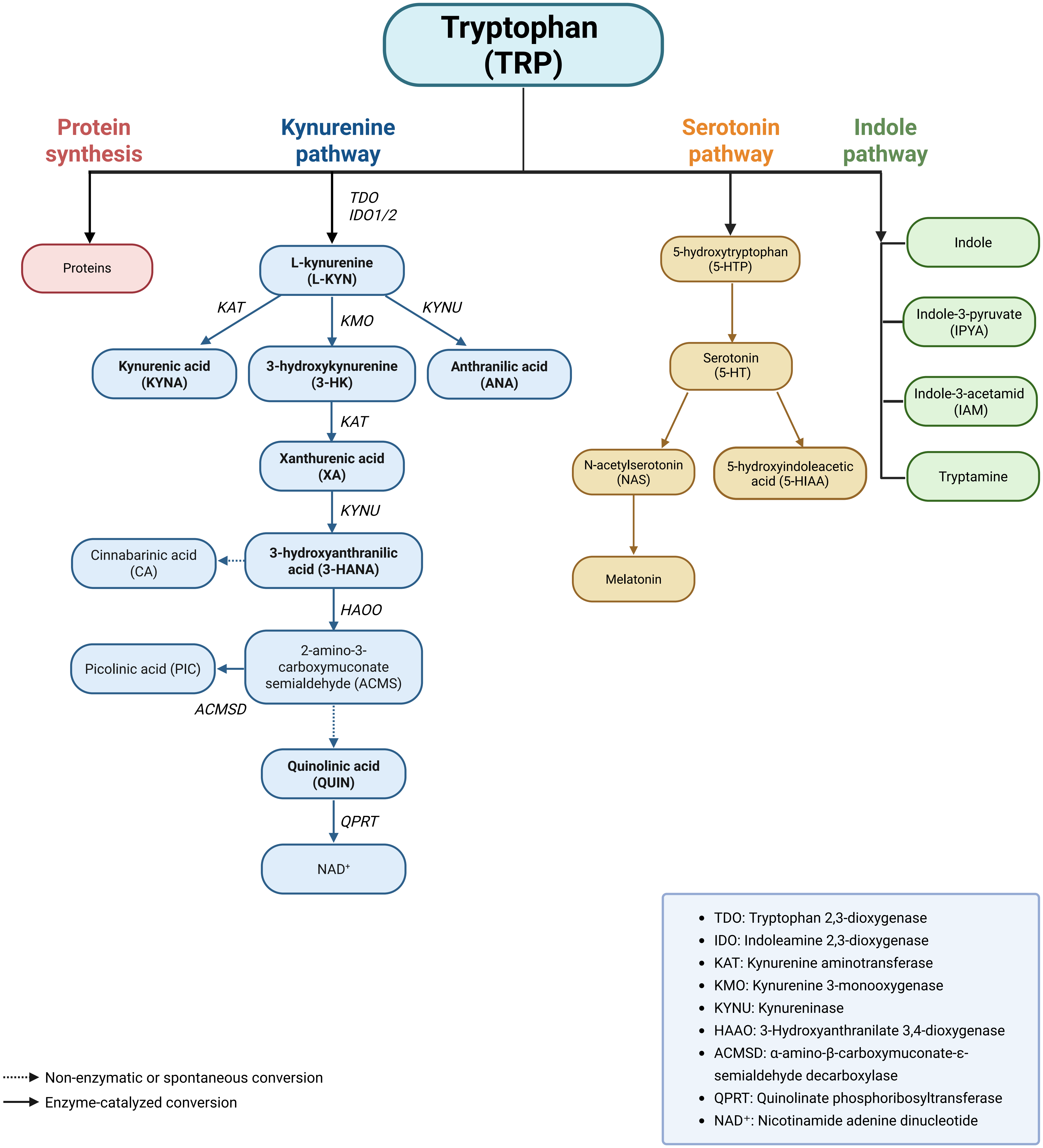

Supplement: Supplementary file 1 [file biomolecules-16-01128-s001.zip › Supplementary Figure S1.pdf]
