# Supplementary material for: Pharmacological Modulation of the Kynurenine Pathway Using PF-04859989 Modulates PACAP Signaling in Migraine-Relevant Trigeminal Sensitization
Source: Biomolecules. 2026 Aug 3;16(8):1128. doi: 10.3390/biom16081128 (PMC13511012; doi:10.3390/biom16081128)
Supplement: Supplementary file 1 [file biomolecules-16-01128-s001.zip › Supplementary Table S1.pdf]

Supplementary Table S1. Summary of kynurenine pathway metabolites quantified by UHPLC-MS

Metabolite	Group	n	Median	IQR	95% CI	Statistical test	Comparison	p-value
**Tryptophan**	control	6	42400	3500	41092.12–44841.21	—	—	—
	CFA	6	53750	4975	51933.68–57666.32	Independent samples t-test	vs control	**<0.001**
	CFA + saline	6	56950	8425	49527.36–63272.64	—	—	—
	CFA + PF-04859989 (16 mg/kg)	6	33950	1325	32881.71–34518.29	One-way ANOVA with Dunnett post hoc test	vs CFA + saline	**<0.001**
	CFA + PF-04859989 (32 mg/kg)	6	58400	6650	53630.81–65435.85	One-way ANOVA with Dunnett post hoc test	vs CFA + saline	0.504
**Kynurenine**	control	6	2525	63	2501.73–2564.93	—	—	—
	CFA	6	2915	100	2858.81–2964.52	Mann–Whitney U test	vs control	**<0.002**
	CFA + saline	6	2910	565	2595.92–3277.41	—	—	—
	CFA + PF-04859989 (16 mg/kg)	6	2540	155	2422.41–2584.26	Mann–Whitney U test	vs CFA + saline	**0.022**

	CFA + PF-04859989 (32 mg/kg)	6	2905	370	2631.96–3418.04	Mann–Whitney U test	vs CFA + saline	0.853
**Kynurenic acid**	Control	6	54.05	3.25	51.94–55.50	—	—	—
	CFA	6	65.05	37.00	35.16–74.27	Mann–Whitney U test	vs control	0.394
	CFA + saline	6	59.60	39.65	37.01–81.66	—	—	—
	CFA + PF-04859989 (16 mg/kg)	6	27.70	2.02	26.50–29.40	Mann–Whitney U test	vs CFA + saline	**0.002**
	CFA + PF-04859989 (32 mg/kg)	6	68.65	12.28	53.09–76.51	Mann–Whitney U test	vs CFA + saline	0.699
**Anthranilic acid**	Control	6	38.95	4.30	35.96–41.74	—	—	—
	CFA	6	33.35	9.85	25.69–36.28	Independent samples t-test	vs control	**0.007**
	CFA + saline	6	35.00	7.57	30.94–41.03	—	—	—
	CFA + PF-04859989 (16 mg/kg)	6	18.80	1.80	17.69–19.78	One-way ANOVA with Dunnett post hoc test	vs CFA + saline	**<0.001**

	CFA + PF 04859989 (32 mg/kg)	6	41.90	6.02	38.76–45.21	One-way ANOVA with Dunnett post hoc test	vs CFA + saline	**0.029**
**3-Hydroxykynurenine**	Control	6	32.10	6.90	28.76–36.24	—	—	—
	CFA	6	33.30	4.80	30.27–36.33	Independent samples t-test	vs control	0.679
	CFA + saline	6	34.60	4.70	31.74–37.39	—	—	—
	CFA + PF-04859989 (16 mg/kg)	6	31.10	2.60	29.34–32.20	One-way ANOVA with Dunnett post hoc test	vs CFA + saline	0.122
	CFA + PF-04859989 (32 mg/kg)	6	35.00	5.20	31.56–39.90	One-way ANOVA with Dunnett post hoc test	vs CFA + saline	0.906
**Xanthurenic acid**	Control	6	43.70	2.53	42.10–45.46	—	—	—
	CFA	6	48.75	33.43	21.83–57.17	Mann–Whitney U test	vs control	0.394
	CFA + saline	6	45.05	26.45	24.97–53.47	—	—	—

	CFA + PF-04859989 (16 mg/kg)	6	24.05	1.77	23.45–25.42	Mann–Whitney U test	vs CFA + saline	0.394
	CFA + PF-04859989 (32 mg/kg)	6	50.75	11.25	35.61–57.49	Mann–Whitney U test	vs CFA + saline	0.240
**3-Hydroxyanthranilic acid**	Control	6	46.25	1.38	45.35–47.71	—	—	—
	CFA	6	42.10	12.48	32.35–45.62	Mann–Whitney U test	vs control	**0.002**
	CFA + saline	6	42.40	29.22	15.45–97.58	—	—	—
	CFA + PF-04859989 (16 mg/kg)	6	38.35	2.80	36.62–39.81	Mann–Whitney U test	vs CFA + saline	0.093
	CFA + PF-04859989 (32 mg/kg)	6	42.30	2.23	41.30–44.54	Mann–Whitney U test	vs CFA + saline	0.818
**Quinolinic acid**	Control	6	284.0	28.0	264.74–303.93	—	—	—
	CFA	6	524.0	560.0	384.80–1015.87	Mann–Whitney U test	vs control	**0.002**
	CFA + saline	6	846.5	865.0	363.27–1386.40	—	—	—

	CFA + PF-04859989 (16 mg/kg)	6	1075.0	60.0	1032.63–1117.37	Mann–Whitney U test	vs CFA + saline	1.000
	CFA + PF-04859989 (32 mg/kg)	6	527.5	354.5	210.82–1250.18	Mann–Whitney U test	vs CFA + saline	0.699

Abbreviations: CFA: Complete Freund's Adjuvant; **UHPLC-MS:** ultra-high-performance liquid chromatography–mass spectrometry; **ANOVA**: analysis of variance; **IQR:** interquartile range; **CI:** confidence interval
