# Supplementary material for: Pharmacological Modulation of the Kynurenine Pathway Using PF-04859989 Modulates PACAP Signaling in Migraine-Relevant Trigeminal Sensitization
Source: Biomolecules. 2026 Aug 3;16(8):1128. doi: 10.3390/biom16081128 (PMC13511012; doi:10.3390/biom16081128)
Supplement: Supplementary file 1 [file biomolecules-16-01128-s001.zip › Supplementary Table S2.pdf]

Supplementary Table S2. Summary of prepropPACAP expression quantified by Western blot analysis

Group	n	Median	IQR	95% CI	Statistical test	Comparison	p-value
control	6	0.3405	0.387	0.222–0.631	—	—	—
CFA	6	0.9030	0.513	0.534–1.202	Independent samples t-test	vs control	**0.016**
CFA + saline	6	0.9985	0.507	0.604–1.217	—	—	—
CFA + PF-04859989 (16 mg/kg)	6	1.7370	0.688	1.158–1.988	One-way ANOVA with LSD post hoc test	vs CFA + saline	**0.001**
CFA + PF-04859989 (32 mg/kg)	6	1.2640	0.295	0.986–1.542	One-way ANOVA with LSD post hoc test	vs CFA + saline	0.052

Abbreviations: CFA: Complete Freund's Adjuvant; **ANOVA:** analysis of variance; **LSD:** least significant difference; **IQR:** interquartile range; **CI:** confidence interval; **preproPACAP**: prepro-pituitary adenylate cyclase-activating polypeptide
